# A Review of Neuroimaging Research of Chinese as a Second Language: Insights from the Assimilation–Accommodation Framework

**DOI:** 10.3390/bs15091243

**Published:** 2025-09-12

**Authors:** Jia Zhang, Xiaoyu Mou, Bingkun Li, Hehui Li

**Affiliations:** 1 School of Psychology and Cognitive Science, Beijing Language and Culture University, Beijing 100083, China; zhangjia@blcu.edu.cn (J.Z.);; 2 Key Laboratory of Language and Cognitive Science (Ministry of Education), Beijing Language and Culture University, Beijing 100083, China; 3 National Key Laboratory of Human Factors Engineering, China Astronaut Research and Training Center, Beijing 100094, China; 4 China Astronaut Research and Training Center, Beijing 100094, China; 5 Center for Brain Disorders and Cognitive Sciences, School of Psychology, Shenzhen University, Shenzhen 518060, China

**Keywords:** L2 Chinese learners, assimilation, accommodation, neuroimaging, universality and specificity

## Abstract

The assimilation–accommodation theory provides a crucial theoretical framework for understanding the neural mechanisms of second language (L2) processing. Chinese characters, as logographic scripts, contain diverse strokes and components with high visual complexity, and their grapheme–phoneme conversion differs fundamentally from alphabetic writing systems. Existing studies have identified unique neural patterns in Chinese language processing, yet a systematic synthesis of L2 Chinese processing remains limited. This review focuses on the brain mechanisms underlying Chinese language processing among L2 learners with diverse native language backgrounds. On the one hand, Chinese language processing relies on neural networks of the native language (assimilation); on the other hand, it recruits additional right-hemisphere regions to adapt to Chinese characters’ visuospatial complexity and grapheme–phoneme conversion strategies (accommodation). Accordingly, this review first synthesizes current brain imaging studies on L2 Chinese processing within this theoretical framework, noting that prevailing paradigms—limited to lexical and sentence-level processing—fail to capture the complexity, hierarchy, and dynamics of natural language. Next, this review examines the application and implications of naturalistic stimuli paradigms in neuroimaging research of L2 Chinese processing. Finally, future directions for this field are proposed. Collectively, these findings reveal neuroplasticity in processing complex ideographic scripts.

## 1. Introduction

The uniqueness of Chinese orthography dictates its neurocognitive specificity. For L2 (second language) Chinese learners, neural substrates differ markedly from those engaged in their native language processing. Against the backdrop of globalization and international education, demand for Chinese language learning continues to grow. The recent integration of cognitive neuroscience methods into research on L2 Chinese learners has shed new light on the physiological and cognitive mechanisms of language learning. Elucidating the neural mechanisms of Chinese as a L2 processing holds significant practical value for optimizing pedagogy. However, despite these advances, systematic reviews that integrate these findings within a coherent theoretical framework remain limited. The present article therefore provides a comprehensive review of neuroimaging research on L2 Chinese processing. Specifically, we synthesize existing evidence within the assimilation–accommodation framework to clarify how learners from different native language backgrounds adapt their neural resources when acquiring Chinese as a second language. Systematic studies on L2 Chinese learners can disentangle cross-linguistic universality and specificity, revealing both the first language (L1) transfer effects and L2-driven neural adaptations. After establishing the scope and purpose of this review, we then introduce the linguistic and cognitive characteristics of Chinese reading and comprehension. This contextual background highlights why the assimilation–accommodation framework is particularly relevant for interpreting the neural mechanisms of L2 Chinese processing.

The essence of reading lies in extracting information from written symbols, with fluent reading achieved through the integration of orthographic, phonological, and semantic processing to bridge spoken and written language (Huber et al., 2018). While reading processes across languages may share universal processes, specificity arises from variations in grapheme–phoneme mapping rules and visual–orthographic characteristics. For instance, Chinese (Sino-Tibetan) and English (Indo-European)—two representative languages with vast native speaker populations—differ markedly. Chinese characters are square-structured (consisting of strokes or radicals that fit into a square-shaped space) with high visual complexity and holistic processing demands, whereas English words follow linear configurations (Li et al., 2022; Wu et al., 2012; Tan et al., 2005). Neuroimaging studies have identified three core reading systems: the left temporo–parietal, temporo–occipital, and inferior/middle frontal regions (Black et al., 2017; Wu et al., 2012; Xia et al., 2016). While brain structure and function are initially genetically determined (Hawrylycz et al., 2015), the functional organization for reading is also modulated by writing systems (Bolger et al., 2005; Tan et al., 2005).

The universality and specificity of reading networks across languages constitute a key question in cognitive neuroscience. Some studies emphasized cross-linguistic universality. For example, Rueckl et al. (2015) demonstrated through semantic categorization tasks that skilled readers of both alphabetic and logographic languages share a common reading network, irrespective of orthographic transparency. Malik-Moraleda et al. (2022) further revealed a universal fronto–temporo–parietal language network across 45 languages from 12 families. Conversely, language-specific mechanisms exist. Chinese phonological processing directly addresses character pronunciation via the left middle frontal gyrus due to weak phonetic cues, whereas English relies on temporo-parietal regions for phonological assembly (Tan et al., 2005). Additionally, Chinese orthographic processing uniquely recruits right-hemisphere regions (e.g., right fusiform gyrus and superior/inferior parietal lobules) for holistic visuospatial analysis (Guo & Burgund, 2010; Tan et al., 2001). It is essential to investigate universality and specificity in language processing among individuals with different native languages, shaped by both genetic and environmental factors. Similarly to studies examining how monolingual speakers process their native language, the bilingual brain exhibits both shared and language-specific neural systems for reading in the first (L1) and second (L2) languages. Examining how distinct languages are represented during bilingual processing provides critical insights into neural reorganization in new linguistic environments.

The assimilation–accommodation framework distinguishes between two neurocognitive strategies in second language learning: assimilation, whereby L2 processing relies on pre-existing neural mechanisms established for the first language, and accommodation, whereby novel neural adaptations emerge to meet the unique demands of the second language (Perfetti et al., 2007). This framework provides a valuable lens for examining how L2 Chinese learners adapt to the typological and scriptural distinctiveness of Chinese. Based on this theoretical foundation, the first research question we propose is as follows: What characteristics of assimilation and accommodation are reflected in the neural mechanisms of L2 Chinese learners with different native language backgrounds during Chinese language processing? Nevertheless, extant neuroimaging research on L2 processing predominantly focuses on isolated lexical or sentence-level processing (Sulpizio et al., 2020), which inadequately reflects the complexity, hierarchy, and dynamics of natural language comprehension. To address this gap, we further concern the following: How might naturalistic paradigms advance our understanding of assimilation–accommodation in L2 Chinese processing? To this end, this review first synthesizes universal and language-specific patterns identified in neuroimaging studies of L2 Chinese learners. It then advocates for the adoption of naturalistic paradigms and advanced methods—particularly Inter-Subject Correlation (ISC) and Inter-Subject Representational Similarity Analysis (IS-RSA)—to overcome limitations of ecological validity. Finally, we interpret these findings through the assimilation–accommodation lens and outline promising directions for future research.

## 2. Screening Criteria and the Assimilation–Accommodation Framework

### 2.1. Screening Criteria

To comprehensively and systematically examine the progress and overall landscape of neuroimaging studies on L2 Chinese processing from the perspective of the assimilation–accommodation theory, this review follows the methodological standards of systematic reviews.

Relevant literature was primarily retrieved through a systematic search of the Web of Science and PubMed databases, covering the period from its inception to June 2025. The search strategy combined keywords across three conceptual domains: (1) research population (i.e., “second language Chinese”, “L2 Chinese”, “bilingual”); (2) research techniques (i.e., “neuroimaging”, “fMRI”); and (3) theoretical framework (i.e., “assimilation”, “accommodation”).

The screening process was independently conducted by two researchers, with disagreements resolved through discussion to ensure objectivity. The procedure consisted of two stages: (1) initial screening of titles and abstracts to remove clearly irrelevant records; and (2) full-text review of the remaining articles based on predefined inclusion criteria. Studies were included if they met all of the following requirements: (a) participants were L2 Chinese learners; (b) neuroimaging techniques were employed to investigate the neural mechanisms of Chinese language processing; (c) the design included key within-group comparisons (i.e., L2 Chinese learners processing L1 vs. Chinese) or between-group comparisons (i.e., L2 Chinese learners vs. native speakers processing Chinese); and (d) reported results specified brain regions relevant to language processing. In addition, studies focusing solely on native speakers processing Chinese or theoretical articles without empirical brain data were excluded. Ultimately, 13 eligible studies were identified.

Given the heterogeneity of study designs, participant characteristics, and outcome measures, conditions for a quantitative meta-analysis were not yet met. Therefore, a qualitative thematic synthesis approach was adopted. Extracted information—including sample characteristics, experimental paradigms, and core findings—was systematically summarized, compared, and integrated. The assimilation–accommodation framework served as the guiding lens for interpreting evidence, identifying patterns of neural adaptation strategies, and analyzing the factors shaping these strategies.

### 2.2. Assimilation–Accommodation Framework

The assimilation–accommodation framework has been widely applied in language cognition research to investigate the neural mechanisms of L2 processing (Perfetti et al., 2007; Sparks, 1995). At its core, the framework explains how L2 learners’ brains adapt to the demands of the target language through two complementary strategies: assimilation, or the recruitment of L1 neural networks, and accommodation, or the adjustment of neural resources to meet the specific demands of the L2.

The neural validity of this framework has been supported by key empirical evidence. For instance, Nelson et al. (2009) used an fMRI passive viewing paradigm to compare Chinese native speakers processing English with English native speakers. The results showed that Chinese native speakers displayed a bilateral fusiform gyrus activation pattern similar to their L1 when processing English, consistent with an assimilation strategy. In contrast, English native speakers exhibited additional recruitment of the right fusiform gyrus to cope with the spatial complexity of Chinese characters, as well as reliance on the left middle frontal gyrus for integrating orthographic, phonological, and semantic information—reflecting an accommodation strategy. This classic finding demonstrates that L2 processing in bilinguals is not only constrained by existing L1 networks but also requires neural reorganization in response to the features of the target language.

Against this background, adopting the assimilation–accommodation framework as the guiding lens of the present review is particularly significant. First, it provides a robust theoretical foundation for integrating diverse empirical findings. Chinese differs systematically from alphabetic languages in orthography (logographic system, visual complexity), phonology (tones, syllable–character mapping), and grammar (Li et al., 2022). Neuroimaging studies of L2 Chinese learners reveal heterogeneous patterns, with some showing strong overlap with L1 processing and others engaging additional brain regions. Without a unifying framework, these results remain fragmented. The assimilation–accommodation perspective, however, allows such findings to be systematized: reliance on L1 networks is interpreted as assimilation, whereas neural reorganization to address Chinese-specific demands is interpreted as accommodation. In this way, the framework transforms disparate findings into a coherent explanatory model. Second, the framework is heuristic, enabling in-depth investigation of the mechanisms driving different adaptation patterns. This review, for example, addresses two central issues: how learners’ native language backgrounds influence the adoption of assimilation versus accommodation strategies, and how different paradigms, particularly naturalistic ones, can extend the applicability of the framework. These considerations also point to promising directions for future research.

In sum, the assimilation–accommodation framework constitutes both the theoretical foundation and the organizing logic of this study. It not only provides a systematic lens for synthesizing existing evidence but also offers explanatory depth, allowing the review to move beyond summarizing “what is” to addressing “why it is,” thereby advancing our understanding of the neural plasticity underlying L2 Chinese acquisition.

## 3. Neuroimaging Research on L2 Chinese Processing

### 3.1. Manifestations of Accommodation Strategies in L2 Chinese Processing

Kim et al. (2016) substantiated the assimilation–accommodation theory. Their study compared Korean-native speakers processing Korean (KK group) and L2 Chinese speakers (KC group), revealing shared and distinct neural patterns. Shared activations included bilateral inferior frontal gyrus and left fusiform gyrus. Divergently, the KK group relied on the left superior temporal gyrus, left supramarginal gyrus and right insula, which are associated with phonemic parsing and articulatory rehearsal, reflecting the phonological processing advantage of alphabetic languages (Price, 2000; Simos et al., 2000). The KC group, however, showed stronger activation in the bilateral middle occipital gyrus, the left superior parietal lobule, and the left middle frontal gyrus (MFG), with the former two supporting the visuospatial analysis of characters and the latter mediating whole-character-to-syllable mapping (Kim et al., 2016; Perfetti et al., 2013; Tan et al., 2005). Additionally, the KC group exhibited enhanced left inferior parietal lobule (IPL) activation, likely due to the delayed phonological access of Chinese requiring short-term phonological storage (Ravizza et al., 2004). Collectively, the KK group relied on a mature L1 “phonological network”, whereas the KC group adopted a “visuospatial-dominant” strategy for Chinese (Kim et al., 2016; Perfetti & Liu, 2005).

Other studies similarly supported accommodation strategies in L2 Chinese learners. Liu et al. (2007) found that English-native learners, after brief training (3–4 days), shifted activation patterns toward native-like processing: Chinese character reading engaged the bilateral MFG, the right fusiform gyrus, and the occipital regions, whereas English word reading relied on the left inferior frontal gyrus (IFG) and other phonological areas. Crucially, the combined pronunciation–meaning (P+M) training group showed stronger left MFG activation than pronunciation-only (P) or meaning-only (M) groups, suggesting its role in “visual form-sound-meaning” integration (Liu et al., 2007). Ng (2008) examined early English–Chinese bilinguals during passive viewing of English/Chinese words and pseudowords. Chinese reading activated the bilateral fusiform gyrus (versus the left-lateralized for English), with the right fusiform gyrus critical for global visual processing (Ng, 2008), implicating its adaptation to Chinese characters’ holistic visual demands. Nelson et al. (2009) further confirmed the right fusiform gyrus involvement in Chinese visuospatial process. Cao and Perfetti (2016) incorporated an imagined writing task alongside passive viewing, revealing that English-native L2 Chinese learners exhibited stronger left MFG activation during Chinese (versus English) processing in both tasks. Characters learned via handwriting elicited higher left MFG activation during reading than those learned via pinyin, which mirrored English-like activation. This suggests L2 Chinese learners recruit writing-related regions (e.g., left MFG) to accommodate the “visual form-meaning-motor” integration demands of Chinese (Cao & Perfetti, 2016). J. Zhao et al. (2012) also reported right occipito-temporal (fusiform and lingual gyrus) activation in L2 Chinese learners, with the alphabetic L1 backgrounds processing both regular and irregular Chinese characters, attributable to the visual complexity of Chinese (J. Zhao et al., 2012). These studies collectively demonstrated that L2 Chinese learners compensate for the unique features of the script by enhancing visuospatial regions (i.e., right occipital regions and superior parietal lobule) and whole-character phonological-addressed areas (i.e., left MFG), aligning with accommodation.

However, Chee et al. (2001) reported divergent findings. In a semantic judgment task with early English–Mandarin bilinguals (acquired before age 5), L2 Chinese learners increased left prefrontal and parietal activation compared to L1, but no right-hemisphere recruitment was observed. This suggests neural resource allocation adjusts to L2 proficiency without right-hemisphere compensation, possibly due to early L2 exposure enabling left-hemisphere specialization for Chinese (Chee et al., 2001).

Furthermore, some studies have directly compared neural activity between L2 Chinese learners and native speakers during Chinese language processing. For instance, Yu et al. (2018) employed a pronunciation verification task of Chinese pseudo-phonograms to systematically examine differences in orthography-to-phonology conversion (OPC) mechanisms. The study found that native Chinese speakers predominantly relied on left-hemisphere networks (e.g., left inferior parietal lobule) for efficient, automated processing, whereas Chinese L2 learners, unable to fully depend on L1 phonological rules, exhibited a more extensive bilateral activation pattern (right frontal and parietal regions). Specifically, L2 learners compensated for phonological processing by recruiting right-hemisphere regions for visuospatial analysis (e.g., right parietal regions integration of character structure) and executive control (e.g., right inferior frontal gyrus suppressing L1 phonological interference) (Abutalebi & Green, 2016; Yu et al., 2018). This bilateral activation extends to more complex semantic processing. Lai et al. (2021) used a semantic judgment task and observed that high-proficiency L2 Chinese learners with alphabetic L1 backgrounds also showed enhanced activation in the bilateral occipital regions (e.g., fusiform gyrus, middle occipital gyrus) and the right superior parietal lobule, indicating reliance on visuospatial compensation for semantic processing (Lai et al., 2021). Similar findings emerge for logographic L1 backgrounds. Huang et al. (2012) studied Japanese native speakers (with over one year of Chinese instruction) during silent reading. While Japanese native speakers shared a left-hemisphere network (inferior frontal gyrus–temporoparietal–ventral premotor cortex–anterior temporal lobe) for both Japanese and Chinese, they additionally activated the bilateral lateral parieto–occipital lobes (LPOLs), a Chinese-specific visual processing region. Compared to native Chinese speakers, Japanese native speakers exhibited weaker activation in the right fusiform gyrus (reflecting underdeveloped visual processing of characters) but elevated activation of the anterior temporal lobe (approaching Chinese native-like levels), suggesting a shift toward Chinese analytic syntactic strategies (Huang et al., 2012). Thus, despite Japanese and Chinese sharing virtually identical morphographic characters, processing Chinese as L2 still requires adaptation to its unique orthographic and syntactic features, mirroring native-like networks with supplemental activation. Collectively, these comparisons of Chinese as L2 and L1 corroborate accommodation strategies in L2 Chinese processing.

### 3.2. Manifestations of Assimilation Strategies in L2 Chinese Processing

In L2 processing, neural activity is also strongly shaped by learners’ L1, reflecting the assimilation strategy. When the processing routines of the L1 align with the demands of the L2, learners tend to transfer and rely on pre-existing L1 neural networks. Kim et al. (2016) not only demonstrated the accommodation effect through a comparison between the KK and KC groups in Section 3.1, but also illustrated the assimilation effect through a comparison between Korean native speakers reading Korean (KK group) and English (KE) groups. The KE group showed greater activation only in the left inferior frontal gyrus relative to the KK group, suggesting highly overlapping networks, which indicates that Korean speakers may process English under the influence of their L1 neural architecture, with a strong tendency to recruit L1-based networks during L2 comprehension. Comparisons with English native speakers (EE group) further confirmed this L1 effect: the KE group exhibited stronger activation in the left precuneus, the left angular gyrus, and the right supramarginal gyrus—regions more engaged in Korean than in English reading (Lee et al., 2004). Such transfer and reuse of L1 neural networks is understandable: as both Korean and English are alphabetic languages, they share core mechanisms of phonological processing (e.g., phoneme analysis, articulatory rehearsal), which provide the basis for assimilation.

More intriguingly, however, this assimilation pattern is not limited to alphabetic–alphabetic L2 learning. Studies of alphabetic L1 learners of Chinese have also demonstrated evidence of assimilation, suggesting that reliance on L1 networks can persist even when the L1 and L2 differ substantially in script, phonology, and linguistic structure. This highlights the pervasive role of assimilation in shaping L2 Chinese processing. For example, Tian et al. (2019) administered a Chinese character naming task to English–Chinese bilinguals, revealing the pivotal role of L1 in shaping L2 neural representations (Tian et al., 2019). The consistency effect in the left inferior frontal gyrus and the supplementary motor area indicated greater phonological effort for inconsistent characters, with these regions typically associated with phonological processing and rehearsal in alphabetic languages (Cornelissen et al., 2009; Veroude et al., 2010). This demonstrates Chinese L2 learners’ reliance on L1-based phonological compensation, aligning with assimilation. J. Zhao et al. (2012) reported analogous findings: Chinese L2 learners with alphabetic L1 backgrounds showed heightened activation in the left inferior parietal lobule and the ventral inferior frontal gyrus for irregular Chinese characters—core regions for assembled phonology in alphabetic systems—suggesting L1-derived sublexical decomposition strategies for Chinese grapheme–phoneme conversion (J. Zhao et al., 2012). Assimilation also occurs with logographic-L1 learners of Chinese. Lin et al. (2024) found that Japanese–Chinese bilinguals predominantly engaged L1-like (Japanese Kanji) networks (left inferior frontal gyrus and middle temporal gyrus) for Chinese language processing, reflecting transferable phonological mechanisms (Lin et al., 2024).

In summary, irrespective of the typological distance between the learners’ native language and Chinese as a second language, L2 processing strategies are modulated by both L1 networks and target-language (Chinese) features. These findings underscore the cerebral flexibility and specificity in processing complex logographic scripts, fully consistent with the assimilation–accommodation framework.

## 4. The Application and Implications of Naturalistic Stimulus Paradigms in L2 Chinese Processing: Perspectives from ISC and IS-RSA Methods

Through a systematic review of the studies, the current neuroimaging research on L2 Chinese processing has primarily employed three types of traditional paradigms. The first involves character- and word-level processing tasks, such as lexical decision, character naming, and phonological/semantic consistency judgment (Tian et al., 2019; J. Zhao et al., 2012; Chee et al., 2001; Lai et al., 2021). The second consists of sentence-level processing tasks, mainly focusing on syntactic judgment and sentence comprehension (Huang et al., 2012). The third relies on passive viewing paradigms, in which participants observe or listen to linguistic stimuli without explicit behavioral tasks (Nelson et al., 2009; Ng, 2008; Liu et al., 2007). By precisely manipulating linguistic variables, these paradigms isolate fundamental processing components such as orthography, phonology, and semantics, thereby providing important evidence for revealing neural mechanisms of assimilation and accommodation. For example, character-naming tasks have revealed neural signatures of L1 phonological transfer to L2 (Tian et al., 2019), while sentence comprehension tasks demonstrate neural reorganization as learners adapt to Chinese syntactic features (Huang et al., 2012).

Despite their contributions, these paradigms face significant limitations in ecological validity. Existing neuroimaging studies on L2 processing largely rely on lexical, semantic, or syntactic judgment tasks, typically using isolated words, phrases, or simple sentences as experimental stimuli (Sulpizio et al., 2020). While these approaches uncover task-specific neural mechanisms, the controlled settings often fail to capture the complexity and dynamism of natural language comprehension. Specifically, they cannot fully reflect (1) the universal and language-specific mechanisms of bilingual language processing in real-world contexts, (2) the individualized neural representations shaped by learners’ experiences, or (3) the neural bases of cross-level semantic integration and contextual construction. To address these limitations, researchers have increasingly turned to naturalistic paradigms (Tang et al., 2023; Zhang et al., 2023), which more closely approximate the demands of complex, ecologically valid language comprehension (Sonkusare et al., 2019; Willems et al., 2020). While Zhou et al. (2021) employed naturalistic stimuli to demonstrate the developmental shift in native Chinese reading from phonology-dominant to visual–semantic network engagement, such paradigms have yet to be systematically applied to L2 Chinese learners.

The adoption of naturalistic paradigms provides a unique opportunity to evaluate the assimilation–accommodation framework under conditions that approximate real-world language processing. During dynamic, continuous comprehension of naturalistic linguistic input, L2 Chinese learners must integrate phonological, lexical, and syntactic information in real time. This process simultaneously evokes assimilation (e.g., relying on L1 orthographic strategies to recognize Chinese characters) and accommodation (e.g., adjusting comprehension strategies in response to tonal or syntactic differences), reflecting a dynamic interplay between pre-existing cognitive structures and adaptive reorganization. Naturalistic paradigms are particularly well suited to capturing these real-time adaptive mechanisms, revealing how the brain flexibly recruits and reorganizes networks during unconstrained language processing.

To fully leverage the potential of naturalistic stimuli in elucidating the neural mechanisms of L2 Chinese processing, analytical methods suited to such paradigms are essential. Traditional General Linear Model (GLM) approaches exhibit notable shortcomings in investigating the neural underpinnings of natural language processing, as they struggle to adequately capture the continuity and dynamic nature of language processing. In contrast, Inter-Subject Correlation (ISC) analysis can effectively reveal group-consistent neural responses during naturalistic stimulus processing (Hasson et al., 2012; Nastase et al., 2019). By examining the temporal synchronization of neural signals across participants exposed to identical naturalistic stimuli, ISC identifies stimulus-driven consistent responses, thereby uncovering group-level universal processing mechanisms. This characteristic makes ISC particularly suitable for exploring the common neural foundations exhibited by L2 Chinese learners during natural language comprehension, providing a powerful tool for validating universal cognitive adaptive processes within the assimilation-accommodation framework. For instance, Zhang et al. (2023) found that higher-proficiency L2 learners exhibited neural synchronization patterns more similar to native speakers in key regions such as the default mode network and lateral prefrontal cortex during natural language processing. This suggests that ISC can capture the progression of L2 learners’ neural processing patterns converging toward native-like patterns (assimilation), offering methodological support for validating the assimilation–accommodation theory in ecologically valid contexts. Furthermore, ISC can be combined with individual difference measures. For example, correlating Chinese proficiency (e.g., scores on the HSK, an acronym for Hanyu Shuiping Kaoshi—the standardized Chinese Proficiency Test that assesses vocabulary knowledge and listening comprehension) with ISC indices may identify brain regions where processing consistency reflects language ability. Such neural synchrony analyses could provide ecologically valid neural markers for individualized language assessment and pedagogical intervention.

Beyond investigating universal mechanisms, ISC can be integrated with other methods to study neural bases of individual differences. Inter-Subject Representational Similarity Analysis (IS-RSA), combining ISC with Representational Similarity Analysis (RSA), examines relationships between behavioral differences and neural representation differences, revealing how individual characteristics (e.g., reading ability, task comprehension) map onto unique neural signatures (Baek et al., 2022; Finn et al., 2020; Jangraw et al., 2023). For L2 Chinese processing, IS-RSA could elucidate whether processing patterns at different linguistic levels (phonology, lexico-semantics, syntax, discourse coherence) vary with learners’ L1 backgrounds or cognitive strategies. For instance, learners from different L1 backgrounds may exhibit distinct neural representation patterns when processing Chinese tones, syntactic structures, or discourse organization—individual differences that IS-RSA can detect while controlling for group-level processing commonalities.

In summary, ISC and IS-RSA methods address both group-level universal mechanisms and individual-specific neural architectures in L2 Chinese processing. Future studies should integrate naturalistic paradigms with longitudinal designs to validate the assimilation–accommodation theory in authentic language contexts and systematically characterize the neural developmental trajectories of L2 Chinese learners.

## 5. Discussion

### 5.1. Applications of the Assimilation–Accommodation in Neuroimaging Research of L2 Chinese Processing

Based on a systematic examination within the assimilation–accommodation theoretical framework, L2 Chinese learners from different native language backgrounds exhibit distinct yet systematic patterns of neural adaptation. Learners whose native language is alphabetic (e.g., English or Korean) adopt a dual-path adaptive strategy when processing Chinese. On the one hand, they rely on their L1 phonological processing network (e.g., the left inferior frontal gyrus and the inferior parietal lobule) for phonemic analysis and rehearsal, reflecting a typical “assimilation” mechanism (Tian et al., 2019; J. Zhao et al., 2012). On the other hand, to accommodate the visuospatial complexity and low transparency of orthography–phonology mappings in Chinese characters, they recruit regions such as the right fusiform gyrus, the middle occipital gyrus, and the left middle frontal gyrus, supporting enhanced visual parsing and holistic character processing, thus demonstrating clear “accommodation” characteristics (Kim et al., 2016; Yu et al., 2018). In contrast, L2 learners whose native language is logographic (e.g., Japanese) tend to reuse neural resources due to similarities in writing systems. The overlap in regions such as the left inferior frontal gyrus and middle temporal gyrus with those engaged in native logograph processing (e.g., Japanese Kanji) highlights a marked “assimilation” advantage (Lin et al., 2024). Nonetheless, the unique orthographic and syntactic features of Chinese still necessitate the recruitment of areas such as the bilateral lateral parieto–occipital lobes and the left anterior temporal lobe for adaptive adjustment, indicating a more limited yet essential “accommodation” process (Huang et al., 2012). Collectively, these findings suggest that the neural mechanisms of L2 Chinese processing are jointly shaped by both native language background and target language properties, thereby supporting the interactive role of assimilation and accommodation at the neural level as posited by the theory.

Crucially, the adoption of naturalistic stimulus paradigms has further advanced our understanding of assimilation–accommodation mechanisms in L2 Chinese processing by leveraging ecologically valid contexts and innovative analytical approaches. First, naturalistic stimuli require learners to integrate cross-linguistic information in real time, thereby authentically revealing the dynamic interplay between reliance on native language strategies (assimilation) and the recruitment of novel neural resources (accommodation). Second, Inter-Subject Correlation (ISC) analysis within such paradigms enables the quantification of group-level neural convergence. For instance, Zhang et al. (2023) identified neural markers of L2 proficiency during naturalistic language comprehension, showing that higher-proficiency learners exhibited more native-like neural synchronization within the default mode network and lateral prefrontal cortex. This evidence suggests that ISC provides a valid neural index of “nativization” in L2 comprehension, thereby empirically grounding the assimilation–accommodation framework in ecologically realistic settings. Finally, the integration of ISC and representational similarity analysis (IS-RSA) allows for the identification of individual differences, enabling precise characterization of the neural strategies learners from diverse L1 backgrounds employ when processing features such as Chinese tones or syntactic structures. This analytic approach captures fine-grained manifestations of “accommodation” across individuals. Overall, naturalistic paradigms, by creating authentic processing contexts and integrating advanced analytical tools, elucidate the neural underpinnings of assimilation–accommodation from both group-level convergence and individual-level adaptation perspectives. This dual vantage point provides a more comprehensive and dynamic explanatory framework for understanding L2 Chinese processing.

### 5.2. Future Directions in Neuroimaging Research with L2 Chinese Processing

Existing studies have primarily examined L2 Chinese processing within the assimilation–accommodation framework, yielding theoretical insights for pedagogy. Future research could advance the field through several innovations. First, regarding participant selection, current studies predominantly examine alphabetic-L1 bilinguals, with few including logographic-L1 bilinguals (e.g., Japanese). Expanding to diverse L1 backgrounds would elucidate how typological distance modulates neural adaptation strategies (Kim et al., 2016). Such work would test the cross-linguistic generalizability of the assimilation–accommodation hypothesis, revealing universal versus language-specific learning mechanisms and informing comprehensive L2 acquisition models.

Building upon Dynamic Restructuring Model (DRM) proposed by Pliatsikas et al. (2020), which systematically explains the structural brain changes accompanying L2 proficiency development, existing studies on L2 Chinese learners have validated this framework. Luo et al. (2006) demonstrated that during the initial stages of Chinese tone learning, processing predominantly relies on right-hemisphere networks (particularly right inferior frontal gyrus), while advanced learners gradually develop left-hemisphere semantic access networks in language regions, evidenced by strengthened left superior temporal gyrus engagement and reduced dependence on right inferior frontal gyrus. This reflects dynamic neural reorganization from right frontal to left temporal dominance during tone processing (Luo et al., 2006). Similarly, Qi et al. (2019) observed enhanced activation in left inferior frontal gyrus and superior parietal lobule after four weeks of Mandarin training in English native speakers, alongside reduced right inferior frontal gyrus activation that correlated with long-term retention, indicating a shift from right-hemisphere acoustic processing to left-hemisphere phonology–semantics integration. These findings suggest progressive neural reorganization with proficiency in Chinese as L2. Notably, DRM and the assimilation–accommodation theory are complementary in explaining L2 Chinese processing mechanisms. DRM provides a stage-specific neural basis for assimilation and accommodation: the initial reliance on right-hemisphere regions corresponds to the “accommodation” of Chinese-specific features in DRM’s early cortical adjustment stage, while the later shift to left-hemisphere networks reflects “assimilation” of native-like processing, aligning with DRM’s advanced structural optimization. In turn, the assimilation–accommodation theory clarifies the functional significance of DRM’s brain changes—right-hemisphere adjustments serve to “accommodate” unadapted Chinese features, and left-hemisphere activation enhancement enables “assimilation” of efficient processing, jointly uncovering the neural logic of L2 Chinese processing.

Based on the complementary relationship between the two, more longitudinal studies could be conducted in the future to systematically examine the changes in brain structure and function of L2 Chinese learners from the initial learning stage to the advanced stage, thereby clarifying the dynamic correspondence between the various stages of the Dynamic Restructuring Model (DRM) and the transition of “assimilation–accommodation” strategies. This would not only provide a more precise neurodevelopmental trajectory for L2 Chinese learning but also offer targeted theoretical guidance for optimizing pedagogical strategies—such as designing stage-specific training programs that align with learners’ neural adaptation status (e.g., emphasizing right-hemisphere-oriented acoustic training in the early stage to facilitate “accommodation,” and promoting left-hemisphere-based phonology-semantics integration practice in the advanced stage to enhance “assimilation”).

Moreover, task type significantly modulates the neurocognitive mechanisms of Chinese reading, with certain brain regions showing stronger responses to specific tasks (e.g., semantic/syntactic tasks) compared to script type (R. Zhao et al., 2017). Current neuroimaging research on L2 Chinese processing predominantly focuses on phonological and semantic levels, lacking investigations of syntactic or discourse processing. Future studies should incorporate diverse task paradigms to compare L1 and L2 processing with L2 Chinese learners more comprehensively. Future studies may integrate naturalistic stimulus paradigms and design multi-level tasks covering phonological, semantic, syntactic, and discourse dimensions to compare the neural mechanisms underlying L1 and L2 processing in L2 Chinese learners. On the one hand, naturalistic stimuli can simulate real-language scenarios, enabling more accurate capture of the dynamic “assimilation-accommodation” processes during syntactic and discourse processing. On the other hand, by leveraging methods suitable for naturalistic stimuli (i.e., ISC and IS-RSA), it is possible to dissociate group-general and individual-specific “assimilation-accommodation” patterns. Analytically, while existing research primarily used activation analyses to identify regional differences, neural distinctions between L1 and L2 processing may lie in unique activation patterns rather than overall response magnitudes (Weaverdyck et al., 2020). Multivariate pattern analysis (MVPA) offers advantages here by detecting language-specific neural representations within shared regions. Although some studies have applied multivariate approaches to bilingual reading (Dong et al., 2021; Nichols et al., 2021; Qu et al., 2019; Xu et al., 2017), neuroimaging research with L2 Chinese learners remains scarce. Future work should employ MVPA to characterize spatial activation patterns. For instance, Lin et al. (2024) found that while Chinese–Japanese bilinguals shared bilateral occipital lobe and fusiform gyrus activation during character processing, their neural patterns diverged significantly in key language regions (left inferior/middle frontal gyrus). MVPA-based analysis has been shown to effectively differentiate the neural activation patterns underlying the processing of Chinese and Japanese Kanji character (Lin et al., 2024).

Finally, while fMRI excels in spatial localization of functional networks on L2 Chinese processing, its poor temporal resolution limits the investigation of rapid information integration. To address this, researchers increasingly combine fMRI with high-temporal-resolution EEG/MEG for spatiotemporal characterization (Baillet, 2017). Multimodal integration holds particular promise for neuroimaging research with L2 Chinese learners. Compared to alphabetic languages, Chinese demonstrated distinct phonological/syntactic/semantic features that likely involve dynamic interactions across neural modules at multiple timescales. Furthermore, L2 learners exhibit time-varying neural reorganization during natural language processing. Simultaneous fMRI-EEG/MEG can capture instantaneous neural responses (e.g., semantic conflicts) and localize their cortical sources, enabling spatiotemporal decoding of language comprehension processes.

## 6. Conclusions

This review synthesized current evidence on the neural mechanisms underlying L2 Chinese processing within the framework of assimilation and accommodation. Analysis of the existing literature identified two predominant patterns of neural adaptation: assimilation, characterized by the continued engagement of native language networks (e.g., left frontal and temporal regions), and accommodation, marked by the recruitment of additional brain regions such as the right fusiform gyrus for visual processing and the left middle frontal gyrus for addressed phonology. These findings underscore the brain’s remarkable plasticity in adapting to the unique cognitive and linguistic demands of Chinese. Future research should emphasize longitudinal and ecologically valid approaches, integrated with multimodal neuroimaging techniques, to more accurately capture the dynamic trajectory of neural reorganization during Chinese L2 acquisition.
